# Mixed-phenotype leukemia with *TCF3::ZNF384* fusion presenting as an isolated mediastinal mass

**DOI:** 10.1007/s00277-024-06042-x

**Published:** 2024-11-12

**Authors:** Wing-Yan Au, Chit Chow, Ka-Fai To, Edmond S.K. Ma, Eugene C.L. Yeung, Wai-Lun Yip, Helen M.H. Chan, Harinder Gill

**Affiliations:** 1 Blood-Med Clinic, Central, Hong Kong; 2 Department of Anatomical and Cellular Pathology, the Chinese University of Hong Kong, Ma Liu Shui, Hong Kong; 3 Department of Clinical and Molecular Pathology, Hong Kong Sanatorium and Hospital, Happy Valley, Hong Kong; 4 Ethics First Medical Practice HK, Harbour City, Hong Kong; 5 Department of Pathology, the Hong Kong Baptist Hospital, Kowloon Tong, Hong Kong; 6 Department of Medicine, Union Hospital, Tai Wai, Hong Kong; 7 Department of Medicine, School of Clinical Medicine, LKS Faculty of Medicine, the University of Hong Kong, Pok Fu Lam, Hong Kong; 8 Department of Medicine, Queen Mary Hospital, Professorial Block, Pokfulam Road, Hong Kong, China

**Keywords:** Mixed-phenotype leukemia, *TCF3::ZNF384*, Myeloid sarcoma, RNA-sequencing, Whole genome sequencing

## Abstract

Acute leukemia with *TCF3::ZNF384* is a distinct type of acute leukemia that present most commonly as B-acute lymphoblastic leukemia or mixed-phenotype acute leukemia (B/myeloid). We report the first case of *TCF3::ZNF384* mixed-phenotype leukemia presenting as isolated extramedullary disease in the mediastinum. Diagnosis using RNA-sequencing and whole genome sequencing on the primary issue is illustrated.

## Dear Editor,

**Fig. 1:**
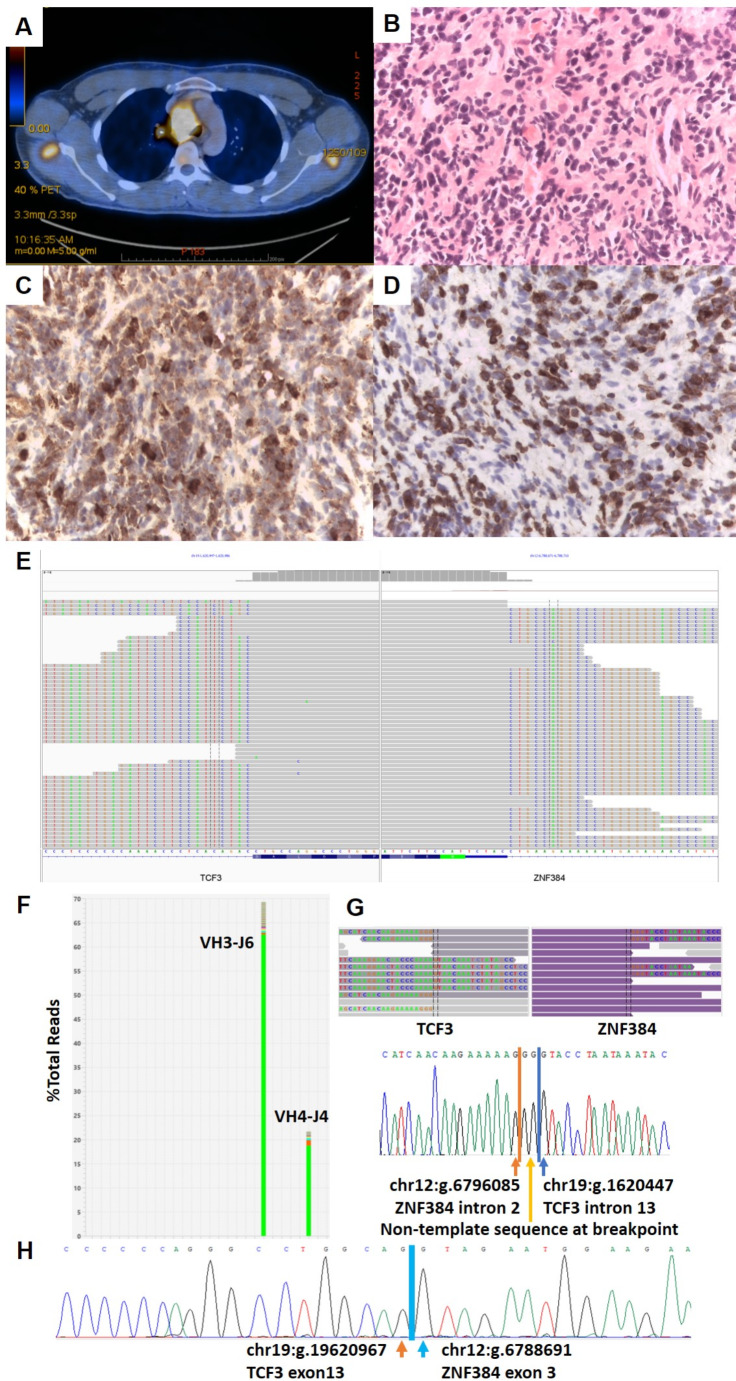
Radiologic, histopathologic and molecular features of mixed-lineage hematolymphoid tumor with *TCF3::ZNF384* fusion. **A**: 18-fluorodeoxyglucouse (18-FDG) positron emission tomography-computerized tomography (PET-CT) showing the hypermetabolic anterior mediastinal tumour with invasion to the aortic arch; **B.** Hematoxylin and eosin (H and E) section showing sheets of medium-sized malignant haematolymphoid cells with dispersed chromatin and inconspicuous nucleoli (400X); **C.** Malignant haematolymphoid cells were positive for myeloperoxidase (MPO) immunostaining (400X); **D.** Malignant haematolymphoid cells were positive for CD79a immunostaining (400X); **E.** Detection of *TCF3::ZNF384* fusion from formalin-fixed paraffin embedded mediastinal biopsy by Illumina Pan Cancer RNA-seq: The split-reads supporting the fusion breakpoints on *TCF3* exon 13 and *ZNF384* exon 3 were shown by Integrative Genomics Viewer (IGV); **F.** Detection of two dominant clonal IgH sequences from formalin-fixed paraffin embedded mediastinal biopsy by LymphoTrack Dx IGH Assays. Top 200 V-J sequence frequencies from IGH FR2 panel were shown; **G.***TCF3::ZNF384* fusion detection by 30X whole-genome sequencing (WGS) (top) and subsequent confirmation with Sanger sequencing (bottom) from the formalin fixed paraffin embedded mediastinal biopsy. Translocation involving *TCF3* (NM_003200.5) intron 13 and *ZNF384* (NM_133476.5) intron 2 was detected by 30X WGS and the corresponding reads were shown by IGV (top). The gene fusion was further confirmed by conventional PCR and Sanger sequencing (bottom). The fusion breakpoint is compatible with the most frequent in-frame *TCF3::ZNF384* Ex13-Ex3 fusion transcript (GRCh37/hg19 reference genome); **H**: Mediastinal biopsy performed at first presentation 18 months ago was analyzed by reverse transcription polymerase chain reaction (RT-PCR) and Sanger sequencing for *TCF3::ZNF384* demonstrating the breakpoint between *TCF3* exon 13 and *ZNF384* exon 3

A 31-year old Chinese man presented in September 2022 with fever and cough. A computerized tomography (CT) showed a 7 cm mediastinal mass. A mediastinal biopsy showed only necrotic tissue. The complete blood count (CBC) was normal. An autoimmune antibody screen showed positivity against extractable nuclear antigen (ENA) and he was treated with oral prednisolone for a presumed autoimmune etiology. There was symptomatic relief with significant reduction in the size of the mediastinal mass on repeated scanning. Eighteen months later, his symptoms recurred together massive hemoptysis. An 18-fluorodeoxyglucose (18-FDG) positron emission tomography CT scan (PET-CT) showed a 7 cm mass with a maximum standard uptake value (SUVmax) of 15, invading the aortic arch (Fig. 1A). An open biopsy showed sheets of medium-sized malignant cells with dispersed chromatin and inconspicuous nuclei (Fig. 1B). The cells are positive for common leucocyte antigen (LCA), myeloperoxidase (Fig. 1C), CD34 and CD43. They also showed expression of CD19 and CD79a (Fig. 1D), but were negative for CD3, CD20, CD10, CD22, Tdt and CD56, with a Ki67 of 10%. Bone marrow (BM) aspiration and biopsy was normal by morphology and no flow cytometry was performed. The patient was treated with daunorubicin (90mg/m^2^/day for 3 days) and cytosine arabinoside (Ara-C) (100mg/m^2^/day for 7 days) “3 + 7” induction therapy with resolution of mediastinal mass. Next generation sequencing (NGS) for a myeloid gene panel of the tumor DNA did not reveal any pathogenic variants. An NGS for a lymphoid gene panel showed the presence of pathogenic *KMT2D* p.L715Pfs*211 and *TBL1XR1* p.A142Yfs*4 variants (Tier III) reported in B-lineage malignancies. An RNA-sequencing panel demonstrated a *TCF3::ZNF384* fusion junction (Fig. 1E). NGS for immunoglobulin heavy chain (IgH) gene rearrangement revealed two dominant clonal FR2 sequences (IGHV3-66_01/IGHJ6_02 at 70.7% and IGHV4-34_02/IGHJ4_02 at 22.8%) and confirmed the presence of clonal B-cells (Fig. 1F). The *TCF3::ZNF384* fusion was confirmed at the genomic level by whole-genome sequencing (WGS) and Sanger sequencing on the extracted tumor DNA (Fig. 1G). The blood and BM at diagnosis showed no detectable disease by DNA breakpoint specific PCR. Retrospective analysis from the first necrotic mediastinal biopsy also showed the presence of *TCF3::ZNF384* fusion (Fig. 1H). The patient received two cycles of high dose Ara-C (3 g/m^2^/dose every 12 h for 4 doses) consolidation. He remained in remission by PET-CT and received an allogeneic hematopoietic stem cell transplantation (HSCT) from a human leucocyte antigen (HLA) identical sibling donor with cyclophosphamide-total body irradiation (Cy-TBI) conditioning.

Leukemia with *TCF3::ZNF384* fusion is a distinct subtype of acute leukemia, with less than 60 reported cases worldwide [1–3]. It was first described in 2002 in pediatric acute lymphoblastic leukemia (ALL) and subsequently in mixed-lineage leukemia (MLL) of B/myeloid lineage [1–3]. There are also repeated reports of lineage switch from B to myeloid under selective pressure when treated [4]. We present a unique case of an adult with mixed lineage leukemia with *TCF3::ZNF384* fusion presenting as an indolent isolated mediastinal mass.

This is the first presentation of leukemia with *TCF3::ZNF384* fusion as an isolated mediastinal tumor. An exact diagnosis was also not possible without molecular means. Since the graft-versus-leukemia effect may not be as potent outside the marrow milieu, incorporation of radiotherapy as part of conditioning prior to HSCT is important.

## Data Availability

No datasets were generated or analysed during the current study.
